# Mechanical Loading Modulates AMPK and mTOR Signaling in Muscle Cells

**DOI:** 10.1021/acs.jproteome.4c00242

**Published:** 2024-08-30

**Authors:** Xin Zhou, Shaochun Zhu, Junhong Li, Andre Mateus, Chloe Williams, Jonathan Gilthorpe, Ludvig J. Backman

**Affiliations:** † Department of Medical and Translational Biology, Faculty of Medicine, Umeå University, 90187 Umeå, Sweden; ‡ Section of Physiotherapy, Department of Community Medicine and Rehabilitation, Faculty of Medicine, Umeå University, 90187 Umeå, Sweden; § Department of Chemistry, Faculty of Medicine, Umeå University, 90187 Umeå, Sweden

**Keywords:** skeletal muscle, exercise adaptation, AMPK, mTOR, mechanical loading, proteomics analysis, protein synthesis, RNA sequencing, mitochondrial biogenesis, ADP/ATP ratio

## Abstract

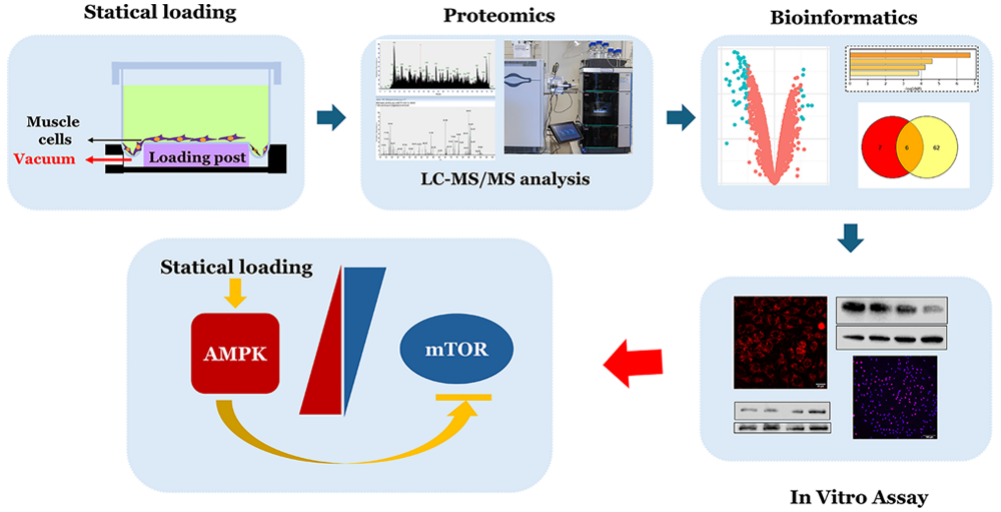

Skeletal muscle adaptation to exercise involves various phenotypic changes that enhance the metabolic and contractile functions. One key regulator of these adaptive responses is the activation of AMPK, which is influenced by exercise intensity. However, the mechanistic understanding of AMPK activation during exercise remains incomplete. In this study, we utilized an in vitro model to investigate the effects of mechanical loading on AMPK activation and its interaction with the mTOR signaling pathway. Proteomic analysis of muscle cells subjected to static loading (SL) revealed distinct quantitative protein alterations associated with RNA metabolism, with 10% SL inducing the most pronounced response compared to lower intensities of 5% and 2% as well as the control. Additionally, 10% SL suppressed RNA and protein synthesis while activating AMPK and inhibiting the mTOR pathway. We also found that SRSF2, necessary for pre-mRNA splicing, is regulated by AMPK and mTOR signaling, which, in turn, is regulated in an intensity-dependent manner by SL with the highest expression in 2% SL. Further examination showed that the ADP/ATP ratio was increased after 10% SL compared to the control and that SL induced changes in mitochondrial biogenesis. Furthermore, Seahorse assay results indicate that 10% SL enhances mitochondrial respiration. These findings provide novel insights into the cellular responses to mechanical loading and shed light on the intricate AMPK–mTOR regulatory network in muscle cells.

## Introduction

Skeletal muscle adaptation to exercise involves a multitude of phenotypic changes that contribute to improved metabolic and contractile functions.^1^ These adaptations include enhanced mitochondrial quality, increased glucose uptake, and improved insulin sensitivity.^2^ An essential player in these adaptive responses is the activation of AMPK (5′-AMP-activated protein kinase).^3^ The activation of AMPK during skeletal muscle contraction is influenced by exercise intensity, with high-intensity exercise resulting in greater AMPK activation compared to low-intensity exercise.^4^ AMPK activation is closely tied to the AMP/ATP ratio, which increases due to significant ATP depletion during exercise. Although the mechanisms by which exercise induces AMPK activation remain to be fully elucidated, the use of in vitro models can provide valuable insights into this process.

To mimic the loading patterns experienced by skeletal muscle in vivo, muscle cells can be subjected to mechanical loading by using the FlexCell Tension system in vitro. However, direct evidence regarding whether mechanical loading induces AMPK activation in vitro is currently lacking. It is demonstrated that AMPK phosphorylation occurs after in situ muscle contraction in rats.^5^ In addition, mechanical loading has been shown to activate AMPKγ3 and upregulate the mammalian/mechanistic target of rapamycin, mTOR signaling.^6^ Unlike active muscle contraction observed in vivo, the FlexCell Tension system induces elongation of muscle cells to mimic the strain of muscle cells in vivo, i.e., passive strain. The potential of this passive strain to activate AMPK remains unknown.

mTOR is a major regulator of mRNA translation/protein synthesis. It functions in two different complexes, mTORC1 and mTORC2, which regulate cell growth and survival, respectively.^7,8^ Current literature suggests that mTORC1 plays a critical role in stimulating mRNA translation/protein synthesis in skeletal muscle.^9^ In skeletal muscle cells, the interplay between two opposing forces, namely, mTORC1 and AMPK, governs muscle adaptation to exercise. mTORC1 promotes muscle growth by mediating the anabolic response to resistance exercise, while AMPK is activated during endurance exercises to activate catabolic processes that ultimately lead to normalization of the AMP/ATP ratio.^10^ A computational model suggests that AMPK stimulation subsequently reduces mTORC1 activation.^11^ Notably, a connecting aspect of exercise to AMPK activation in skeletal muscle is that exercise induced upregulation of AMPK signaling and downregulation of mTOR signaling.^12^ Consequently, exploring the interaction between mTOR and AMPK in muscle cells following various intensities of mechanical loading in vitro is of particular interest.

In this study, we aimed to elucidate the signaling pathways activated in muscle cells subjected to varying static load intensities. Utilizing the FlexCell Tension system, we conducted proteomic analyses and supplementary validation experiments. Our results indicate that mechanical loading modulates RNA metabolism in a dose-dependent manner through the interaction between AMPK and mTOR signaling pathways. Additionally, observed alterations in mitochondrial biogenesis in response to varying loading intensities may underlie the observed AMPK activation.

## Materials and Methods

### Cell Culture

L6 myoblasts from passages 4–7 were cultured in T-175 flasks (Sarstedt, #83.3912.002) in Dulbecco’s modified Eagle medium (DMEM; Thermo Fisher Scientific, no. 31966021) containing l-alanyl-l-glutamine (GlutaMAX) and 10% fetal bovine serum (FBS; Thermo Fisher Scientific, #10500064). Prior to experiments, the FBS concentration was reduced to 1% to minimize its inherent impact on cell proliferation and migration. For proteomics analysis, the medium was switched to SkBM-2 Basal Medium (SkGM-2 μ_B_, Lonza, #CC-3246) supplemented with SkGM-2 SingleQuotsTM without FBS (SkGM-2 SQ, Lonza, #CC-3244), including 0.1% human epidermal growth factor (hEGF), 0.1% dexamethasone, 2% l-glutamine, and 0.1% gentamicin/amphotericin-B (GA). Cells were cultured until 90–100% confluency before loading L6 cells exhibited elevated expression of myosin heavy chain 1 (Myh1) and myogenin (Myog), with a marked reduction in myogenic differentiation 1 (Myod1) expression, when cultured in 1% FBS and SkBM-2 Basal Medium for 24 h as compared to 10% FBS. The increase in Myh1 and Myog, both markers of muscle differentiation, alongside the decrease in Myod1, a marker of proliferating muscle cells, indicate that the cells were undergoing myotube differentiation (Figure S1A). However, despite these molecular changes, no significant morphological alterations were detected during the differentiation process (Figure S1B).

### Mechanical Loading and Drug Treatments

The FlexCell Tension System (Flexcell International Corporation, FX5000, USA) was used to generate mechanical loading of the adherent muscle cells. Cells were seeded on the membranes at a density of 3 × 10^5^ cells per well and cultured until 90–100% confluency before loading. FlexCell plates were placed on 25 mm diameter round equibiaxial loading posts. The flexible-bottomed membranes of the FlexCell plates were stretched by vacuum suction. The muscle cells received static mechanical loading (SL) of 2%, 5%, and 10%. The loading protocol was as follows: 1 h SL followed by 2 h rest period; this SL and rest period was repeated three times before a resting period of 6 h. The protocol was repeated for 24 h. By adding rest intervals and applying mechanical strain for intervals, stimulus adaptation is avoided and the mechanical sensitivity of cells is maintained.^13^ No significant alterations in cell death were observed following the loadings, as determined by lactate dehydrogenase (LDH) assays; data are shown in Figure S2.

For rapamycin (Merck, #53123-88-9) treatment, cells were cultured with 1–3 μM rapamycin for 24 h. For Compound C (Merck, #171260) treatment, cells were cultured with 5 μM Compound C for 24 h with/without SL. For Pim1/AKK1-IN-1 (MCE, # HY-10371) treatment, cells were cultured with/without 1 μM Pim1/AKK1-IN-1 for 24 h with 10% SL.

### Lactate Dehydrogenase Activity Assay

A lactate dehydrogenase (LDH) colorimetric assay kit (Abcam, Cambridge, UK) was used on cell culture medium according to the manufacturer’s instructions. Briefly, culture medium was collected and centrifuged at 2000 rcf at 4 °C for 3 min to remove cell debris. The medium was stored at −80 °C until all groups were collected. Upon thawing, 50 μL of the medium was combined with 50 μL of the provided reaction mixture from the kit. Subsequently, the samples were incubated for 60 min at 37 °C in darkness. LDH activity was determined by measuring the absorbance at 450 nm by using a plate reader.

### Proteomics Analysis

#### Sample Preparation

Medium was switched to SkBM-2 Basal Medium (SkGM-2 μ_B_, Lonza, #CC-3246) supplemented with SkGM-2 SingleQuotsTM without FBS (SkGM-2 SQ, Lonza, #CC-3244), including 0.1% human epidermal growth factor (hEGF), 0.1% dexamethasone, 2% l-glutamine, and 0.1% gentamicin/amphotericin-B (GA), before mechanical loading. The medium was chosen for proteomics analysis since its compound is well-defined, avoiding interferences from FBS due to its complex nature. Samples were collected and digested into peptides using a modified SP3 protocol.^14,15^ Briefly, cell pellets were resuspended in lysis buffer (2% SDS, 20 mM TCEP) and boiled at 95 °C for 10 min. SpeedBeads magnetic carboxylate modified particles (Sigma-Aldrich, beads A hydrophilic, cat. no. GE45152105050250; beads B hydrophobic, cat. no. GE65152105050250,) were combined with a ratio of 1:1 v/v and washed using LC-MS water four times. Then the beads were mixed with each sample in binding buffer (50% ethanol and 2.5% formic acid in final) and incubated with shaking at 500 rpm for 15 min at room temperature (RT). Then they were transferred into one filter plate (0.22 μm, Sigma-Aldrich, part. no. MSGVN2210). The unbound fraction was removed by centrifugation at 1000 rcf. Beads were retained on the filter and washed with 70% ethanol four times. Trypsin was mixed with digestion solution (100 mM HEPES pH 7.5, 5 mM chloroacetamide, 1.2 mM TCEP) and added to each sample (1 μg of trypsin was used for 25 μg of protein) on the plate. Samples were digested overnight at RT with shaking at 500 rpm. Flowthrough containing peptides was collected with centrifugation at 1000 rcf. Ten microliters of 2% DMSO was added to beads for eluting bound peptides and pooled with the previous flowthrough. Peptides were desalted by the Oasis HLB plate (Waters, catalog no. 186001828BA) using the factory protocol and then dried by speed vac.

#### LC-MS/MS

Dried peptides were dissolved with 0.1% formic acid in water. Then, 1 μg of peptides from each sample was introduced to MS using the Vanquish Neo instrument (Thermo Scientific). The trapping column was PEPMAP NEO C18 (5 μm particle size, 300 μm × 5 mm, Thermo Scientific). The analytical column was nano EaseTM M/Z HSS C18 T3 (100 Å, 1.8 μm particle size, 75 μm × 250 mm, Waters). Total length of 2 h for separation and elution was performed with a gradient of mobile phase A (water and 0.1% formic acid) to 8% B (80% acetonitrile and 0.1% formic acid) over 4 min and to 27% B over 87 min, then rise to 80% B in 0.1 min and hold for 4 min, finally to 2% B in 30 s, and finally column equilibration was performed.

Data acquisition on an Exploris 480 instrument (Thermo Scientific) was carried out using a data dependent method. Survey scans covering the mass range of 375–1500 were acquired at a resolution of 120,000, RF lens of 40%, and normalized automatic gain control (AGC) of 300%. Maximum cycling time of 2 s was used to control the number of precursors for tandem-MS/MS (MS2) analysis. Charge states include 2–6 charges. Dynamic exclusion was set to exclude the previously selected precursors for 35 s. MS2 scans were acquired at a resolution of 15,000 (at *m*/*z* 200), with an AGC target value of auto. The isolation window was 1.4 *m*/*z*. HCD fragmentation was induced with a normalized collision energy (NCE) of 30. Isotopes were excluded for the MS2 analysis.

#### Data Analysis

Raw data was searched against the *Rattus norvegicus* UniProt FASTA (proteome identifier [ID] UP000002494) using FragPipe (version 18), and label-free quantification was achieved using the LFQ-MBR workflow. Proteins identified from contaminants and decoys were removed. Only proteins that were quantified in more than one replicate in each group were retained for further analysis. R (ver. 4.2.2) was used for statistics analysis and volcano plots. To reduce technical variation, data was normalized using the vsn package.^16^ Protein differential expression was evaluated by using the limma package. Differences in protein abundances were statistically determined using the Student’s *t* test moderated by Benjamini–Hochberg’s method.

Pathway and process enrichment analysis for differentially expressed proteins was conducted using Metascape (https://metascape.org/gp/index.html#/main/step1). The analysis was performed with *R. norvegicus* specified as the input species and analyzed accordingly. Expression analysis was employed to expedite the process. Pathway and process enrichment analyses utilized several ontology sources, including GO Biological Processes, KEGG Pathway, Reactome Gene Sets, and WikiPathways. The entire genome’s gene set served as the background for enrichment analysis.

### RNA Extraction and qRT-PCR

Extraction of mRNA was performed using the RNA extraction kit (Qiagen, Venlo, Netherlands, #74106) according to the manufacturer’s instructions. Subsequently, a high-capacity cDNA reverse transcription kit (Thermo Fisher, Waltham, MA, USA) was used to reverse transcribe RNA into cDNA. To determine the gene expression, TaqMan Gene Expression Assays (Applied Biosystems, Carlsbad, CA, USA) were used. cDNA was run using a ViiA7 Real-Time PCR system and analyzed with its software (Applied Biosystems, Carlsbad, CA, USA). Gene expression was measured by a TaqMan Gene Expression Assay (Applied Biosystems, Carlsbad, CA, USA) and calculated by the 2^–ΔΔCt^ method. All probes used for real-time PCR (Applied Biosystems, Carlsbad, CA, USA) are summarized in Table 1. Rpl13a was used as the reference gene for normalization.

**Table 1:** All Probes Used for Real-Time PCR

gene name	gene symbol	assay ID
mitochondrially encoded cytochrome C oxidase I	Mt-co1	Rn03296721_s1
mitochondrially encoded NADH:ubiquinone oxidoreductase core subunit 6	Mt-nd6	Rn03296815_s1
mitochondrially encoded NADH:ubiquinone oxidoreductase core subunit 4	Mt-nd4	Rn03296781_s1
inner mitochondrial membrane peptidase subunit 1	Immp1l	Rn01514368_m1
nadh:ubiquinone oxidoreductase complex assembly factor 2	Ndufaf2	Rn01489818_g1
peptidylprolyl isomerase (cyclophilin)-like 4	Ppil4	Rn00452692_m1
myogenin	Myog	Rn00567418_m1
myosin heavy chain 1	Myh1	Rn01751056_m1
myogenic differentiation 1	Myod1	Rn00598571_m1
Rpl13a	Rpl13a	Rn00821946_g1

### RNA/Protein Synthesis Assay

5-Ethynyl uridine (EU) (1 mM final) or l-homopropargylglycine (HPG) (50 μM final) was added to the culture medium for 1 h to label nascent RNA or protein, and then the cells were fixed and permeabilized as previously described.^17^ EU labeling of RNAs was detected using the Click-iT RNA Imaging Kit (Life Technologies, cat. #C10639). HPG labeling of proteins was detected using the Click-iT HPG Alexa Fluor 594 Protein Synthesis Assay Kit (Life Technologies, cat. #C10429), following the manufacturer’s protocol. The intensity ratio of foci to DAPI was quantified. Ten spots were randomly selected from each of three biological replicates using DAPI staining, and the corresponding EU/HPG signal intensity was measured.

### Western Blot

Cells were freeze–thawed and further lysed in RIPA (radioimmunoprecipitation) lysis buffer (Thermo Fisher, Waltham, MA, USA) supplemented with protease and phosphatase inhibitor cocktail (Sigma, St. Louis, MO, USA, #P1860). Total protein concentration was determined with a BCA assay (Thermo Fisher, Waltham, MA, USA). Samples containing 20 μg of protein were separated on SDS–polyacrylamide gels and transferred to PVDF or NC membranes (Thermo Fisher, Waltham, MA, USA). Membranes were blocked in 5% bovine serum albumin in TBST for 1 h before staining with primary antibodies overnight at 4 °C. After washing, the membranes were stained with HRP-conjugated secondary antibodies for 1 h at RT before incubation with ECL solution and then analyzed in an Odyssey Fc Dual-Mode Imaging System (LI-COR Biotechnology, Nebraska, USA). β-Actin was used to normalize the target protein expression. All antibodies used are summarized in Table 2.

**Table 2:** Antibodies Used for Immunostaining and Western Blot

antibody	company	code	applications	molecular weight (kDa)
phospho-AMPK (Thr172)	Cell Signaling	2535S	WB	62
AMPKα	Cell Signaling	2532	WB	62
phospho-mTOR (Ser2448) (D9C2)	Cell Signaling	5536S	WB	289
SRSF2	Thermo Fisher	PA5-92037	WB	35
p70(S6K)	Proteintech	14485-1-AP	WB	70
phospho-p70(S6K)	Proteintech	28735-1-AP	WB	70
β-actin	Cell Signaling	4967	WB	42
antirabbit IgG HRP-linked	Cell Signaling	7074	WB	

### MMP Assay

MMP was examined by assessing the TMRM (Thermo Fisher Scientific). After SL, cells were incubated with 20 nmol/L TMRM (1 h, 37 °C) and Hochest stain 33258 (30 min, 37 °C) in the dark. The membrane of the FlexCell plates was then cut and transferred to 6-well plates. The membrane was washed twice tenderly with PBS, and then 500 μL of culture medium was added on top of the membrane to maintain cell viability. A Leica Thunder Widefield fluorescence microscope was utilized for analysis.

### ADP/ATP Ratio

First, 1 × 10^6^ cells were resuspended in 10 μL of 1x PBS and transferred into a white flat-bottom 96-well plate for ADP/ATP assay. The ADP/ATP ratio was determined using the bioluminescence-based ADP/ATP assay kit (Sigma, #MAK135), following the manufacturer’s instructions. Luminescence readings were recorded using a Synergy HT reader (BioTek). ADP/ATP ratios were calculated by normalizing the ATP level to the corresponding ADP level in each well to mitigate variations attributable to differences in cell number between wells.

### Live-Cell Imaging

Cellular morphology and MMP was assessed in real-time using the Incucyte S3 Live-Cell Analysis System (Sartorius, Ann Arbor, MI, USA). Cells were subjected to SL for 1 h with the presence of 20 nM TMRM. Afterward, the FlexCell plate membrane was cut and transferred to 6-well plates and placed in the Incucyte System and the cell morphology and TMRM signaling were visualized continuously for 6 h. The software was adjusted to obtain nine images per well every 1 h over the 6 h period.

### Seahorse Cellular Stress Assays

To evaluate changes in mitochondrial function, Seahorse XFe96 Cell MitoStress Tests (Agilent technologies, Santa Clara, CA) were performed according to the manufacturer’s protocol. Twenty-four hours prior to the assay, 96-well XFe96 cell culture plates were coated with poly-d-lysine (PDL, 50 μg/mL, Sigma-Aldrich) for 1 h at RT. The PDL was then removed, and the wells were washed once with PBS and subsequently incubated in the presence of collagen type 1 (0.01%, Sigma-Aldrich) overnight. Sixteen hours prior to the assay, Seahorse XFe96 sensor cartridges were hydrated with sterile water and stored in a 37 °C non-CO_2_ incubator. On the day of the assay, the collagen type 1 was aspirated from the culture plates, and 60,000 cells, which had been subjected to either 24 h of 10% SL or unloaded control, were plated in DMEM GlutaMAX media with 1% FBS. The cells were allowed to settle and attach for 8 h prior to the replacement of the cell culture growth medium with MitoStress assay medium, consisting of low buffered pH 7.4 DMEM (Sigma-Aldrich) supplemented with glutamine (2 mM, ThermoFisher Scientific), glucose (10 mM ThermoFisher Scientific), and pyruvate (1 mM, ThermoFisher Scientific). Sterile water in XFe96 sensor cartridges was replaced with Seahorse calibration media and together with the cell culture microplate was incubated in a non-CO_2_ incubator at 37 °C for 1 h prior to the assay. Seahorse inhibitory compounds, oligomycin (1 μM, Sigma-Aldrich), carbonyl cyanide 4-(trifluoromethoxy) phenyl-hydrazone (FCCP, 2 μM, Sigma-Aldrich), and rotenone/antimycin A (0.5 μM each, Sigma-Aldrich) were prepared in assay media and injected into the injection ports of XFe96 sensor cartridges prior to assay start. Oxygen consumption rate (OCR) and extracellular acidification rate (ECAR) were reported as absolute rates (pmol/min for OCR and mpH/min for ECAR). Data were exported from the Seahorse XFe96 Extracellular Flux Analyzer into Seahorse XF Report Generator software.

### Statistics

Data were analyzed by using GraphPad Prism 7 (GraphPad Software, San Diego, CA) software. Student’s *t* test was used for statistical analysis between two groups. One-way analysis of variance (ANOVA) with Tukey’s multiple comparison (post hoc) test was performed in comparisons between more than two groups. Differences were considered statistically significant at a *p*-value of <0.05. All experiments were repeated successfully at least three times. All experimental samples were prepared in triplicate (*n* = 3).

## Results

### Proteomic Analysis Unveils Disparate Myoblast Responses to Mechanical Stimuli

In order to systematically investigate the cellular alterations induced by mechanical stimuli, we undertook a proteomics analysis of muscle cells. Cells were subjected to three distinct static loading (SL) conditions, 2%, 5%, and 10%, for a duration of 24 h with intervals of rest in between. Subsequently, cell lysates were obtained for proteomics analysis, resulting in the identification of a total of 6087 proteins. The quantitative outcomes are provided in the Supporting Information.

**Figure 1:**
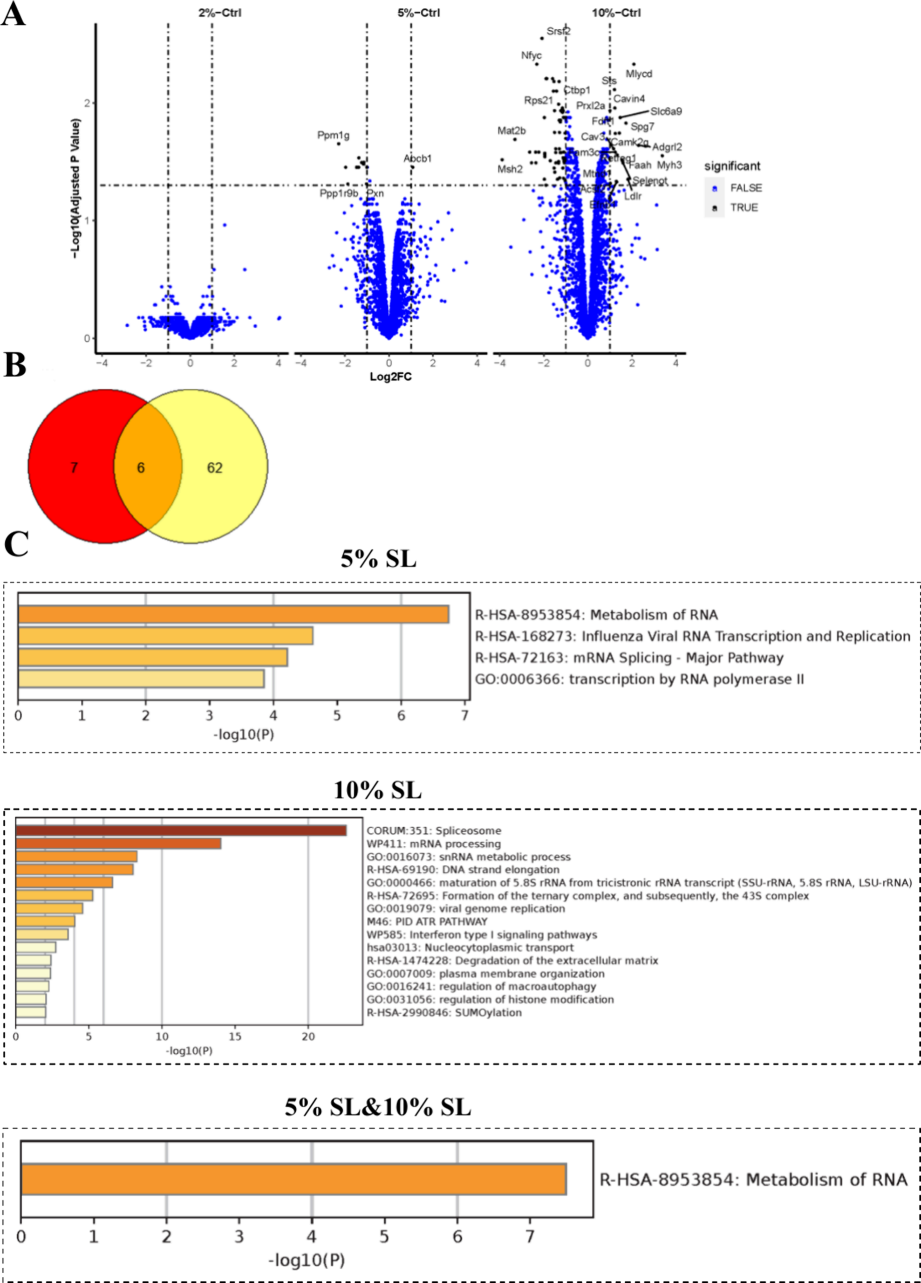
Impact of static loading (SL) on intracellular protein expression in muscle cells. (A) The volcano plot presents the differential expression of proteins between the control and SL groups of 2%, 5%, and 10% at 24 h. Blue dots indicate proteins without statistical significance, whereas black dots represent proteins that are statistically significant (*n* = 3). (B) The Venn diagram depicts the overlap of protein identifications between the 5% and 10% SL groups, highlighting shared protein alterations. (C) Pathway and process enrichment analysis using Metascape reveals the enrichment of signaling pathways in 5% and 10% SL. Notably, there is convergence in the signaling pathways associated with RNA metabolism between the 5% and 10% SL.

When only proteins with a minimum of 2-fold change as compared to the control condition were considered, the 2% SL did not induce any quantitative changes. Conversely, in response to 5% SL, 12 proteins were downregulated, while one protein, ATP binding cassette subfamily C member 4 (ABCC4), was upregulated. Notably, a more pronounced response was observed in muscle cells following 10% SL, with a total of 68 proteins being affected, with 58 proteins downregulated and 10 proteins upregulated (Figure 1A). Of particular interest, six proteins exhibited reduced expression levels in both the 5% and 10% SL groups (Figure 1B). Detailed information regarding these proteins, namely, ribosomal protein SA (RPSA), positive cofactor 4 (SUB1/PC4), transcription elongation factor SPT5 (SUPT5H), serine and arginine rich splicing factor 2 (SRSF2), 40S ribosomal protein S21 (RPS21), and peptidylprolyl isomerase like 4 (PPIL4), is listed in Table S1. Interestingly, these identified proteins play crucial roles in various steps of the gene expression pathway. Specifically, SUB1 and SUPT5H are involved in promoting RNA transcription and elongation, while SRSF2 is necessary for pre-mRNA splicing. RPSA and RPS21 function as core components of the 40S ribosomal subunit, facilitating mRNA scanning and initiation of protein synthesis. Lastly, PPIL4 accelerates protein folding processes. Pathway and process enrichment analysis revealed that the enriched terms associated with 5% and 10% SL predominantly converged on the metabolism of RNA (Figure 1C).

### Statical Loading of 5% and 10% Induces Inhibition of RNA and Protein Synthesis

**Figure 2:**
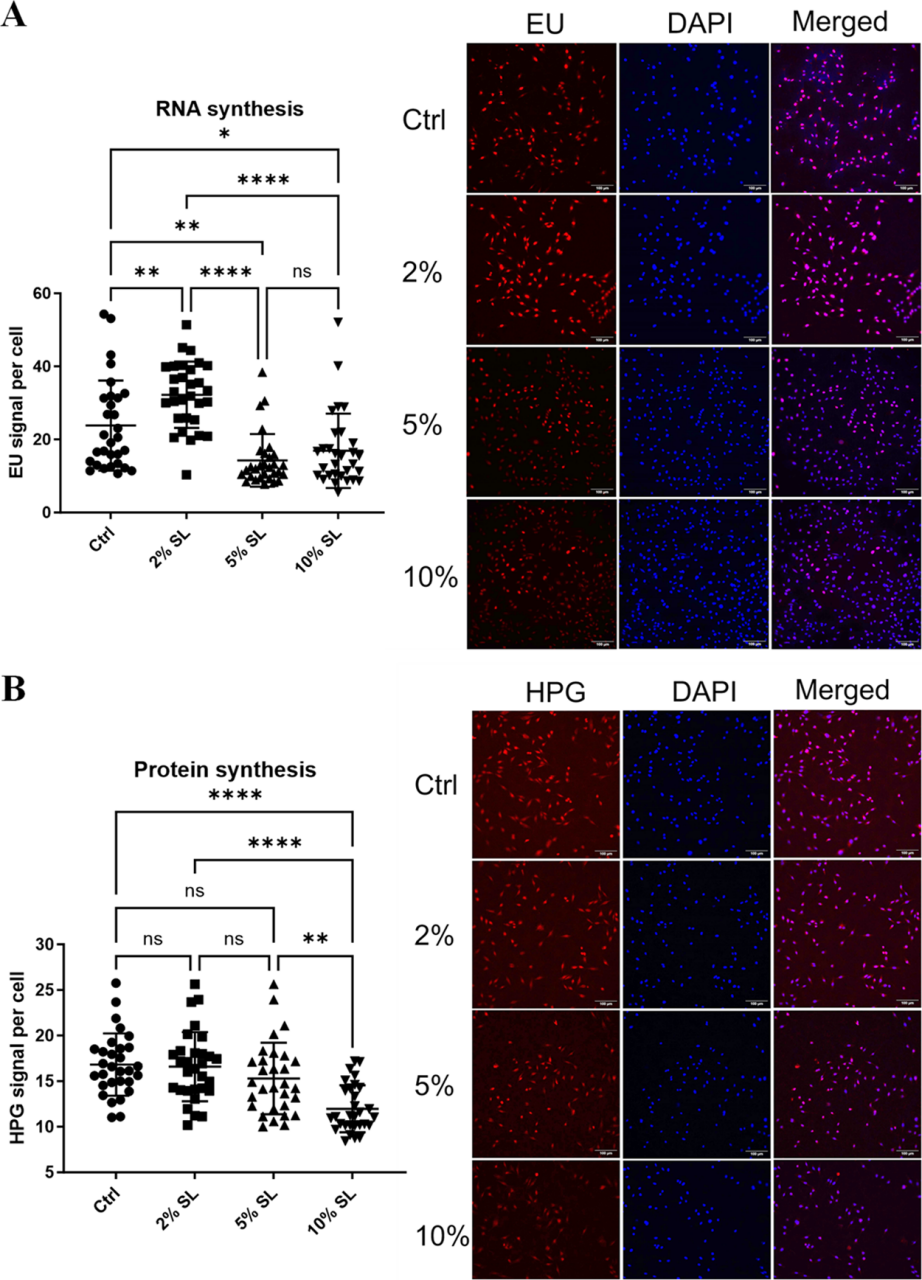
Reduced RNA and protein synthesis by 5% and 10% static loading (SL). (A) Visualization and quantification of RNA synthesis in muscle cells following SL using a Click-iT imaging kit. Quantitative data are presented on the left panel and representative foci images are displayed on the right panel (*n* = 3). (B) Visualization and quantification of protein synthesis in muscle cells following SL using a Click-iT imaging kit. Quantitative data are shown on the left panel and representative foci images are presented on the right panel (*n* = 3). Each dot color represented the result of one of 10 randomly chosen foci from three independent experiments. The data are presented as mean ± standard deviation. Statistical significance is indicated as *p < 0.05, **p < 0.01, and ****p < 0.0001.

Given the involvement of the identified proteins in RNA metabolism and protein synthesis, we proceeded to investigate whether these alterations led to a disruption in RNA and protein synthesis. To address this, we employed the Click-iT kit to assess RNA and protein synthesis in response to SL. While 2% SL resulted in an increase in RNA synthesis as compared to the control, both 5% and 10% SL led to a significant reduction in RNA synthesis (Figure 2A). Similarly, protein synthesis was prominently reduced in muscle cells subjected to 10% SL as compared to both 2% and 5% (Figure 2B).

### 10% Statical Loading Suppresses the mTOR Pathway via AMPK Activation

**Figure 3:**
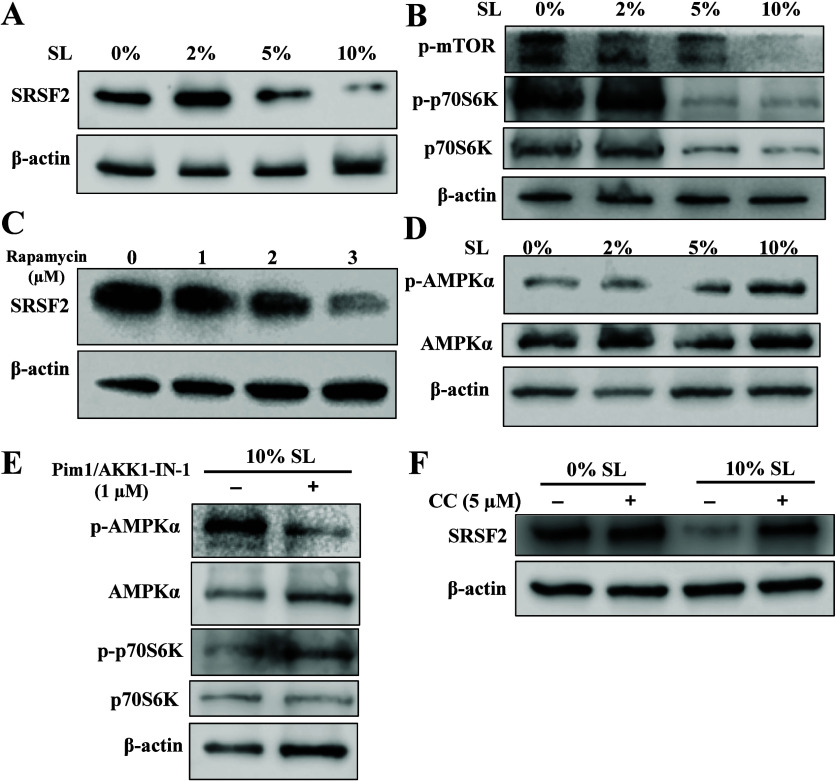
SL suppresses the mTOR pathway via AMPK activation. (A) Decreased expression of SRSF2 in muscle cells in response to increased intensity of SL. (B) Reduced expression of p-mTOR (Ser2448) and p-p70S6K (Ser371) with increased intensity of SL. (C) Muscle cells treated with 1–3 μM rapamycin for 24 h. The results exhibit a dose-dependent reduction in SRSF2 expression. (D) Increased expression of pAMPKα (Thr172) in muscle cells following SL. (E) Pim1/AKK1-IN-1 pretreatment abolished AMPK phosphorylation and rescued p70S6K phosphorylation in loaded muscle cells. (F) Effect of 5 μM Compound C (CC) treatment on SRSF2 expression in muscle cells exposed to 10% SL. Muscle cells were incubated with or without CC for 24 h. The addition of CC rescued the expression of SRSF2 in muscle cells subjected to 10% SL. Actin served as a loading control. Representative immunoblots are shown (*n* = 3).

The top 10 significantly altered proteins following 5% and 10% SL are provided in Tables S1 and S2. Notably, both SUB1 and SRSF2 are ranked as the top two proteins in the 5% and 10% SL groups. Although we encountered difficulties in obtaining an antibody for SUB1 blotting, we present evidence that the expression of SRSF2 is reduced following SL in a dose-dependent manner (Figure 3A), suggesting its potential as a marker for muscle cell response to SL.

The mTOR pathway, known to regulate cell growth and metabolism in response to mechanical loading,^18^ plays a critical role in ribosome biogenesis and protein synthesis regulation.^19^ Therefore, it is reasonable to investigate whether SL affects mTOR signaling. The expression of p-mTOR exhibited an intensity-dependent pattern, with reduced levels observed in 2% and 5% compared to the unloaded control and almost diminished expression in 10%. A similar expression pattern was observed for phosphorylation of the protein S6 kinase (p70S6K), a key indicator of mTOR activation (Figure 3B). Interestingly, treatment with rapamycin, a known mTOR inhibitor, dose-dependently reduced the level of SRSF2 expression (Figure 3C). These findings confirm that SRSF2 is regulated by mTOR in response to SL.

Additionally, we observed that AMPK signaling was activated in response to increased SL, as evidenced by elevated levels of pAMPKα in muscle cells following 5% and 10% SL (Figure 3D). AMPK activation requires LKB1 phosphorylation. Even though we did not find suitable antibodies to blot pLKB1, our data showed that AMPK activation induced by 10% SL was markedly reduced by the addition of LKB1 inhibitor Pim1/AKK1-IN-1 (Figure 3E), suggesting the important role of LKB1 in mediating mechanical loading-induced AMPK activation. Furthermore, we observed that treatment with Pim1/AKK1-IN-1 in 10% SL led to a rescue of p70S6K expression. This finding provides additional confirmation of the interplay between AMPK signaling and the mTOR pathway. To further explore the relationship between AMPK and SRSF2 expression, we utilized Compound C, a specific AMPK inhibitor, which rescued the inhibitory effect of SL on SRSF2 expression (Figure 3F). These data suggest that a higher intensity of SL inhibits the mTOR pathway through AMPK activation. Our finding is consitent with the literature showing that AMPK activation suppressed mTORC1 activity.^20^

### Statical Loading Elevates mtRNA Expression, ADP/ATP Ratio, Mitochondrial Membrane Potential, and Mitochondrial Respiration

**Figure 4:**
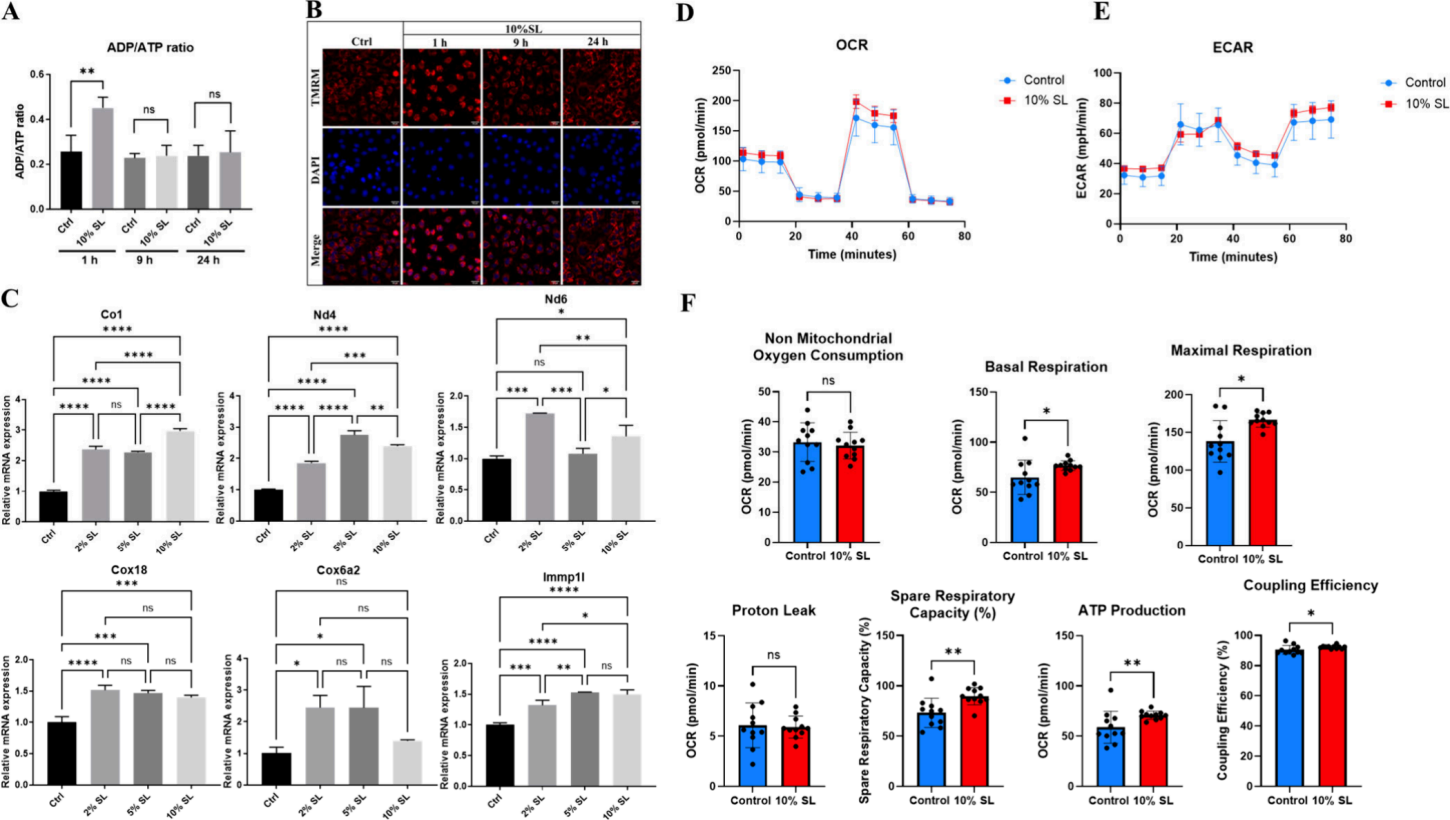
SL induced changes in parameters related to mitochondrial biogenesis. (A) ADP/ATP ratio measured at 1, 9, and 24 h following 10% SL. Significant increase of ADP/ATP ratio was seen at 1 h after 10% SL (*n* = 3). (B) Mitochondrial membrane potential was visulized by TMRM staining. Hoechst stain 33258 was used to label the nucleus. Representative images are shown at each indicated time point (*n* = 3). (C) mRNA expression of mitochondrial proteins encoded by mtDNA (upper panel) and nuclear DNA (lower panel) 24 h after 10% SL (*n* = 3). (D) Oxygen consumption rate (OCR) of control (blue) and 10% SL (red). Oligomycin, FCCP, and rotenone/antimycin A were sequentially added to measure various parameters of mitochondrial respiration (*n* = 3). (E) Extracellular acidification rate (ECAR) of control (blue) and 10% SL (red). Oligomycin, FCCP, and rotenone/antimycin A were sequentially added to assess glycolytic flux (*n* = 3). (F) Quantification of mitochondrial function parameters, including nonmitochondrial oxygen consumption, basal respiration, maximal respiration, proton leak, spare respiratory capacity, ATP production, and coupling efficiency (*n* = 3). The data are presented as mean ± standard deviation. Statistical significance is indicated as *p < 0.05, **p < 0.01, ***p < 0.001, and ****p < 0.0001.

AMPK serves as a crucial sensor of the cellular energy status, becoming activated in response to elevated AMP and ADP levels. To investigate the dynamics of AMPK activation in muscle cells following 10% SL, we monitored the time course of the ADP/ATP ratio. Notably, a rapid increase in ADP/ATP ratio was observed 1 h after applying 10% SL, which subsequently returned to basal levels at 9 and 24 h (Figure 4A). Considering the pivotal role of mitochondria in ATP generation, we examined the parallel changes in the mitochondrial membrane potential. Intriguingly, we detected significant changes in mitochondrial membrane potential (MMP) 1 h after 10% SL, which exhibited a gradual recovery over time (Figure 4B), as measured by TMRM staining. Given that mitochondrial positioning and morphology are known to be regulated by the cytoskeleton,^21^ we employed real-time live imaging to assess the morphological changes following 1 h of 10% SL. We observed immediate cellular morphology shrinkage, which subsequently returned to normal within 6 h, in concurrence with MMP (Supporting Videos S1, S2, and S3). The intricate relationship between the cytoskeleton and energy metabolism prompted us to investigate the potential link between mechanical loading-induced changes in the MMP and the observed shift in the ADP/ATP ratio.

Given the retrospective role of AMPK in stimulating mitochondrial biogenesis, we examined the transcriptional activity of six key genes involved in the electron transport chain (ETC) 24 h after SL (Figure 4C). Quantitative PCR (qPCR) analysis revealed an overall upregulation of mitochondrial DNA (mtDNA)-encoded genes after SL, including Co1, Nd6, and Nd4, as well as nuclear DNA (nDNA)-encoded genes, including Cox18, Ndufaf2, and Cox6a2. Among the tested genes, Mt-nd4, Mt-nd6, and Ndufaf are critical components of NADH:ubiquinone oxidoreductase (mitochondrial complex I), while Cox18, Cox6a2, and Co1 are essential components of cytochrome C oxidase (mitochondrial complex IV). Notably, a general increase in mtRNA expression was observed for all six genes tested, except for Nd6 after 5% SL and Cox6a2 after 10% SL. These results strongly suggest the activation of mitochondrial biogenesis, potentially mediated by AMPK signaling activation.

To further study the effects of SL on mitochondria, a Seahorse XF analyzer was used. The OCR profile shows three key phases of mitochondrial respiration: basal respiration, maximal respiration induced by FCCP, and nonmitochondrial oxygen consumption after addition of rotenone/antimycin A. A comparison of the OCR profile between the control and 10% SL group is illustrated in Figure 4D. Basal respiration and maximal respiration were significantly higher in the 10% SL compared to controls (Figure 4F). There was also a significantly higher level of spare respiratory capacity, ATP production, and coupling efficiency in the 10% SL compared to controls (Figure 4F). Glycolytic activity, as indicated by the ECAR profile, showed similar patterns in response to metabolic stressors between groups (Figure 4E). No significant differences were observed between groups in nonmitochondrial oxygen consumption and proton leak (Figure 4F). Taken together, these results indicate that 10% SL enhances mitochondrial respiration, as evidenced by increased basal and maximal respiration, higher ATP production, and improved coupling efficiency. The increase in spare respiratory capacity suggests that the 10% SL group may enhance the mitochondria’s ability to respond to energetic demands. No significant changes in nonmitochondrial oxygen consumption and proton leak suggest that the observed increases in respiration are due to enhanced mitochondrial function rather than an increase in nonspecific oxygen consumption or mitochondrial damage.

## Discussion

To the best of our knowledge, no study has investigated the proteomic changes in L6 cells after in vitro mechanical loading. The observed intensity-dependent responses of L6 cells to mechanical loading substantiate the reliability of the FlexCell system as a tool for investigating mechanical responses in vitro. Our proteomic profiling offers valuable insights into the distinct responses of cells to different intensities of mechanical stimuli, emphasizing the significance of the AMPK–mTOR signaling pathway. Noteworthy, our proteomics analysis identified four proteins, namely SUB1 (PC4), SRSF2, RPSA, and RPS21, that have been previously associated with mTOR signaling.^19^ SUB1 is an upstream regulator of mTOR, as it inhibits the deacetylation activity of Sin3-HDAC, thereby activating mTOR signaling.^22^ Unfortunately, suitable antibodies for detecting SUB1 in rat muscle cells were unavailable, necessitating further investigation in future studies. Given that the mTOR pathway governs the synthesis of ribosomal components, including ribosomal proteins (RPs),^19^ such as RPSA and RPS21, it is plausible that these proteins are regulated by mTOR. In-depth investigations are required to establish a direct connection between mTOR signaling and RPs. We further showed that SRSF2 expression followed the pattern of mTOR activation and were reversely regulated by the AMPK pathway. These findings suggest that SRSF2 expression is regulated by the AMPK–mTOR signaling axis.

An in vivo study using global phosphoproteomic analysis of human skeletal muscle discerningly unveiled a similar pattern in the AMPK–mTOR crosstalk (Figure 2C in^12^). A similar result is reported by Nelson et al. when employing phosphoproteomics in human, rats, and mice.^5^ In addition to their findings, our findings also underscore the crosstalk between AMPK and mTOR signaling. Additionally, the activation of AMPK was accompanied by dynamic alterations in MMP and upregulation of pivotal genes involved in mitochondrial biogenesis. It is noteworthy that, despite an overall inhibition of RNA synthesis following 10% SL, most expressions of mtRNA were augmented after SL. This outcome strongly supports the notion of enhanced mitochondrial biogenesis after SL. Together with the observed cellular morphological changes, mitochondrial membrane potential, and enhanced mitochondrial capacity, our findings suggest adaptive responses in mitochondria, working in conjunction with the cytoskeleton, to the mechanical loading. These results provide valuable insights into the adaptive responses of muscle cells to mechanical stimuli and their implications for cellular energy metabolism.

The rat L6 cell line is an excellent model system for studying muscle biogenesis in vitro. In this study, we loaded L6 cells under very low serum or serum-free conditions for 24 h. When mononucleate L6 myoblast cells reach confluence in a culture plate, they can transform into multinucleate myotubes through serum starvation.^23^ Indeed, we found that L6 cells underwent myotube transformation, as indicated by significantly reduced Myod1 expression and increased Myh1 and Myog expression. However, the formation of multinucleated cells was not observed due to the short treatment duration. In future studies, we will explore the translation of these findings to human myotubes, despite potential challenges arising from heterogeneity due to incomplete differentiation or variable fusion rates.

## Conclusion

Overall, our study presents novel findings about the proteomic changes and signaling pathways involved in muscle cells in responses to mechanical loading in vitro. This study contributes to an understanding of the molecular mechanisms underlying muscle adaptation to exercise and highlights the interplay between AMPK and mTOR signaling in this process.

## Data Availability

The mass spectrometry proteomics data have been deposited to the PRIDE Archive (http://www.ebi.ac.uk/pride/archive/) via the PRIDE partner repository with the data set identifier PXD050925.

## Supporting Information Available

Figure S1, phenotype changes under different culture conditions; Figure S2, quantification of LDH content in the medium from loaded L6 cells; Figure S3, densitometry data of Western blot analysis corresponding to Figure 3; Table S1, downregulated genes in both 5% and 10% loading groups in muscle cells; and Table S2, top 10 downregulated genes after 5% and 10% loading in muscle cells (PDF)
Summary of the differentially regulated proteins in L6 cells after SL (XLSX)
Video S1, live imaging of L6 cells 1–6 h after SL (MP4)
Video S2, the TMRM signals of L6 cells 1–6 h after SL (MP4)
Video S3, merged live imaging and TMRM signals of L6 cells 1–6 h after SL (MP4)
Original uncropped and unadjusted images of Western blots (PDF)

## References

[ref1] Ferraro E.; Giammarioli A. M.; Chiandotto S.; Spoletini I.; Rosano G. Exercise-Induced Skeletal Muscle Remodeling and Metabolic Adaptation: Redox Signaling and Role of Autophagy. Antioxid Redox Sign 2014, 21 (1), 154–176. 10.1089/ars.2013.5773. PMC4048572 24450966

[ref2] Spaulding H. R.; Yan Z. AMPK and the Adaptation to Exercise. Annu. Rev. Physiol. 2022, 84, 209–227. 10.1146/annurev-physiol-060721-095517. 35143330 PMC8919726

[ref3] Hargreaves M.; Spriet L. L. Skeletal muscle energy metabolism during exercise. Nat. Metab 2020, 2 (9), 817–828. 10.1038/s42255-020-0251-4. 32747792

[ref4] Kjobsted R.; Hingst J. R.; Fentz J.; Foretz M.; Sanz M. N.; Pehmoller C.; Shum M.; Marette A.; Mounier R.; Treebak J. T.; Wojtaszewski J. F. P.; Viollet B.; Lantier L. AMPK in skeletal muscle function and metabolism. Faseb J. 2018, 32 (4), 1741–1777. 10.1096/fj.201700442R. 29242278 PMC5945561

[ref5] Nelson M. E.; Parker B. L.; Burchfield J. G.; Hoffman N. J.; Needham E. J.; Cooke K. C.; Naim T.; Sylow L.; Ling N. X.; Francis D.; Norris D. M.; Chaudhuri R.; Oakhill J. S.; Richter E. A.; Lynch G. S.; Stockli J.; James D. E. Phosphoproteomics reveals conserved exercise-stimulated signaling and AMPK regulation of store-operated calcium entry. EMBO J. 2019, 38 (24), e102578 10.15252/embj.2019102578. 31381180 PMC6912027

[ref6] Riedl I.; Osler M. E.; Björnholm M.; Egan B.; Nader G. A.; Chibalin A. V.; Zierath J. R. AMPKγ3 is dispensable for skeletal muscle hypertrophy induced by functional overload. Am. J. Physiol-Endoc M 2016, 310 (6), E461 10.1152/ajpendo.00387.2015. PMC4796264 26758685

[ref7] Dancey J. mToR signaling and drug development in cancer. Nat. Rev. Clin Oncol 2010, 7 (4), 209–219. 10.1038/nrclinonc.2010.21. 20234352

[ref8] Dufour M.; Dormond-Meuwly A.; Demartines N.; Dormond O. Targeting the Mammalian Target of Rapamycin (mTOR) in Cancer Therapy: Lessons from Past and Future Perspectives. Cancers (Basel) 2011, 3 (2), 2478–500. 10.3390/cancers3022478. 24212820 PMC3757428

[ref9] Goodman C. A. Role of mTORC1 in mechanically induced increases in translation and skeletal muscle mass. J. Appl. Physiol (1985) 2019, 127 (2), 581–590. 10.1152/japplphysiol.01011.2018. 30676865

[ref10] Mounier R.; Lantier L.; Leclerc J.; Sotiropoulos A.; Foretz M.; Viollet B. Antagonistic control of muscle cell size by AMPK and mTORC1. Cell Cycle 2011, 10 (16), 2640–6. 10.4161/cc.10.16.17102. 21799304

[ref11] Sadria M.; Layton A. T. Interactions among mTORC, AMPK and SIRT: a computational model for cell energy balance and metabolism. Cell Commun. Signal 2021, 19 (1), 57 10.1186/s12964-021-00706-1. 34016143 PMC8135154

[ref12] Hoffman N. J.; Parker B. L.; Chaudhuri R.; Fisher-Wellman K. H.; Kleinert M.; Humphrey S. J.; Yang P. Y.; Holliday M.; Trefely S.; Fazakerley D. J.; Stöckli J.; Burchfield J. G.; Jensen T. E.; Jothi R.; Kiens B.; Wojtaszewski J. F. P.; Richter E. A.; James D. E. Global Phosphoproteomic Analysis of Human Skeletal Muscle Reveals a Network of Exercise-Regulated Kinases and AMPK Substrates (vol 22, pg 922, 2015). Cell Metabolism 2015, 22 (5), 948–948. 10.1016/j.cmet.2015.10.004. PMC4635038 26437602

[ref13] Sheng R. W.; Jiang Y. J.; Backman L. J.; Zhang W.; Chen J. L. The Application of Mechanical Stimulations in Tendon Tissue Engineering. Stem Cells International 2020, 2020, 1 10.1155/2020/8824783. PMC7532391 33029149

[ref14] Hughes C. S.; Foehr S.; Garfield D. A.; Furlong E. E.; Steinmetz L. M.; Krijgsveld J. Ultrasensitive proteome analysis using paramagnetic bead technology. Mol. Syst. Biol. 2014, 10 (10), 757 10.15252/msb.20145625. 25358341 PMC4299378

[ref15] Hughes C. S.; Moggridge S.; Müller T.; Sorensen P. H.; Morin G. B.; Krijgsveld J. Single-pot, solid-phase-enhanced sample preparation for proteomics experiments. Nat. Protoc 2019, 14 (1), 68 10.1038/s41596-018-0082-x. 30464214

[ref16] Huber W.; von Heydebreck A.; Sultmann H.; Poustka A.; Vingron M. Variance stabilization applied to microarray data calibration and to the quantification of differential expression. Bioinformatics 2002, 18 (Suppl. 1), S96–104. 10.1093/bioinformatics/18.suppl_1.S96. 12169536

[ref17] Zhou X.; Li J.; Giannopoulos A.; Kingham P. J.; Backman L. J. Secretome from In Vitro Mechanically Loaded Myoblasts Induces Tenocyte Migration, Transition to a Fibroblastic Phenotype and Suppression of Collagen Production. Int. J. Mol. Sci. 2021, 22 (23), 13089 10.3390/ijms222313089. 34884895 PMC8657858

[ref18] Hornberger T. A. Mechanotransduction and the regulation of mTORC1 signaling in skeletal muscle. Int. J. Biochem Cell B 2011, 43 (9), 1267–1276. 10.1016/j.biocel.2011.05.007. PMC3146557 21621634

[ref19] Mayer C.; Grummt I. Ribosome biogenesis and cell growth: mTOR coordinates transcription by all three classes of nuclear RNA polymerases. Oncogene 2006, 25 (48), 6384–6391. 10.1038/sj.onc.1209883. 17041624

[ref20] Alers S.; Loffler A. S.; Wesselborg S.; Stork B. Role of AMPK-mTOR-Ulk1/2 in the Regulation of Autophagy: Cross Talk, Shortcuts, and Feedbacks. Mol. Cell. Biol. 2012, 32 (1), 2–11. 10.1128/MCB.06159-11. 22025673 PMC3255710

[ref21] Solomon T.; Rajendran M.; Rostovtseva T.; Hool L. How cytoskeletal proteins regulate mitochondrial energetics in cell physiology and diseases. Philos. Trans R Soc. Lond B Biol. Sci. 2022, 377 (1864), 20210324 10.1098/rstb.2021.0324. 36189806 PMC9527905

[ref22] Chen L.; Liao F. Y.; Wu J.; Wang Z. W.; Jiang Z. Y.; Zhang C.; Luo P.; Ma L.; Gong Q.; Wang Y.; Wang Q.; Luo M.; Yang Z. Y.; Han S. Q.; Shi C. M. Acceleration of ageing via disturbing mTOR-regulated proteostasis by a new ageing-associated gene PC4. Aging Cell 2021, 20 (6), e13370 10.1111/acel.13370. 33957702 PMC8208792

[ref23] Jiang Y.; Singh P.; Yin H.; Zhou Y.-X.; Gui Y.; Wang D.-Z.; Zheng X.-L. Opposite roles of myocardin and atrogin-1 in L6 myoblast differentiation. J. Cell Physiol 2013, 228 (10), 1989–95. 10.1002/jcp.24365. 23526547 PMC4278347

