# Supplementary material for: Mechanical Loading Modulates AMPK and mTOR Signaling in Muscle Cells
Source: J Proteome Res. 2024 Aug 30;23(10):4286–95. doi: 10.1021/acs.jproteome.4c00242 (PMC11459513; doi:10.1021/acs.jproteome.4c00242)
Supplement: Supplementary file 1 — pr4c00242_si_001.pdf [file pr4c00242_si_001.pdf]

Supplemental data for

Mechanical loading modulates AMPK and mTOR signaling in muscle cells

Xin Zhou [1,†], Shaochun Zhu [3,†], Junhong Li [1,2], Andre Mateus [3], Chloe Williams [1], Jonathan Gilthorpe [1], and Ludvig J. Backman [1,2,*]

1 Department of Medical and Translational Biology, Faculty of Medicine, Umeå University, 90187 Umeå, Sweden

2 Section of Physiotherapy, Department of Community Medicine and Rehabilitation, Faculty of Medicine, Umeå University, 90187 3 Department of Chemistry, Faculty of Medicine, Umeå University, 90187 Umeå, Sweden

*** Correspondence: ludvig.backman@umu.se (L.J.B.)**

† These authors contributed equally to this work.

Table of contents:

1. **Figure S1. Phenotype changes under different culture conditions.**
2. **Figure S2. Quantification of LDH content in the medium from loaded L6 cells.**
3. **Figure S3. Densitometry data of Western blot analysis corresponding to Figure 3.**
4. **Table S1. Down-regulated genes in both 5% and 10% loading groups in muscle cells.**
5. **Table S2. Top 10 down-regulated genes after 5% and 10% loading in muscle cells.**
6. **Supporting Material S1; Summary of the differentially regulated proteins in L6 cells after SL.**
7. **Videos showing live-imaging of L6 cells 1-6 hours after SL. Supporting Material S2: TMRM signal; Supporting Material S3: Supporting Material S4: Merged.**
8. **Supporting Material S5; Original uncropped and unadjusted images of Western blots**

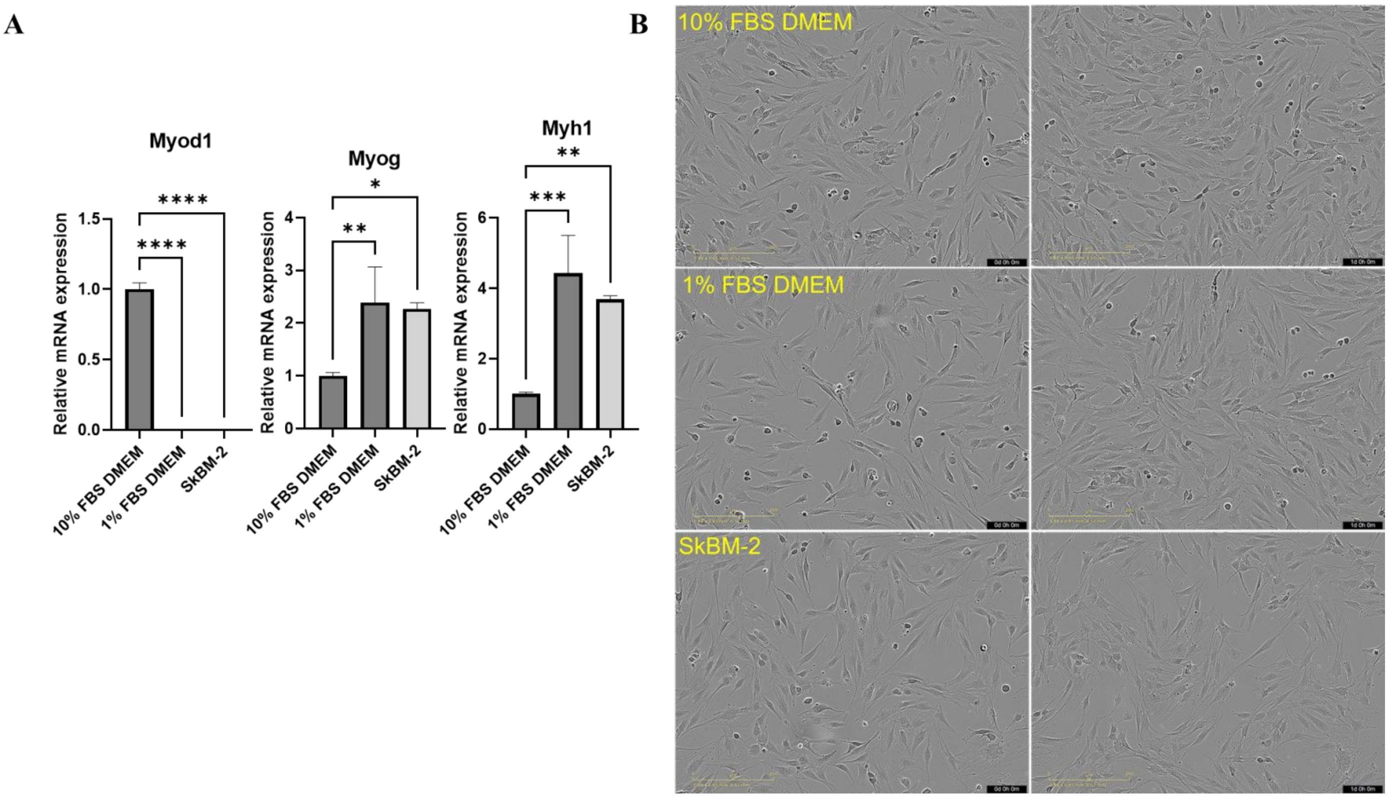

Figure S1. Phenotype changes under different culture conditions. L6 cells were cultured in 10% FBS DMEM, 1% FBS DMEM and SkBMTM-2 Basal Medium for 24 hours, respectively. (A) Expression of myoblast marker Myod1 and myotube markers Myh1 and Myog in different culture conditions. mRNA expression was measured by RT-qPCR. (B) Representative images of muscle cells cultured under varying conditions. Images were captured at the initial time point (0 hours) (left panel) and again after 24 hours (right panel) of culture. Statistical significance is indicated as * $p < 0.05$, ** $p < 0.01$.

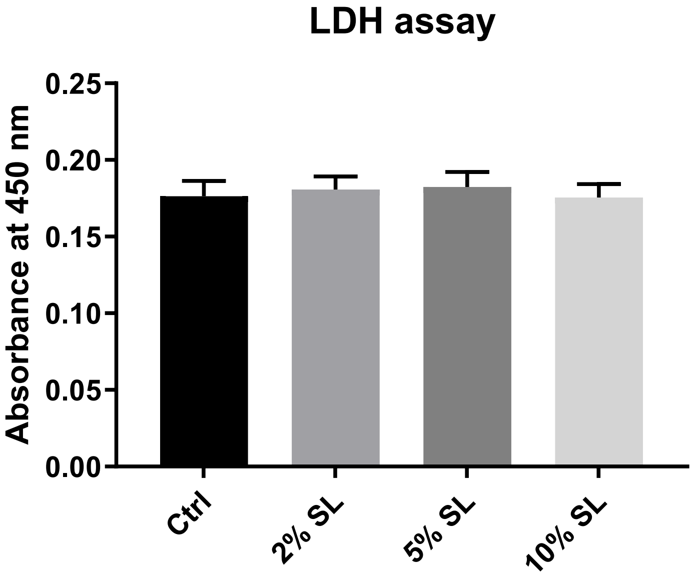

Figure S2. Quantification of LDH content in the medium from loaded L6 cells. L6 cells were subjected to static loading (SL) at 2%, 5%, and 10% intensity. Following the loading, the culture media from each condition were collected and analyzed using the LDH assay.

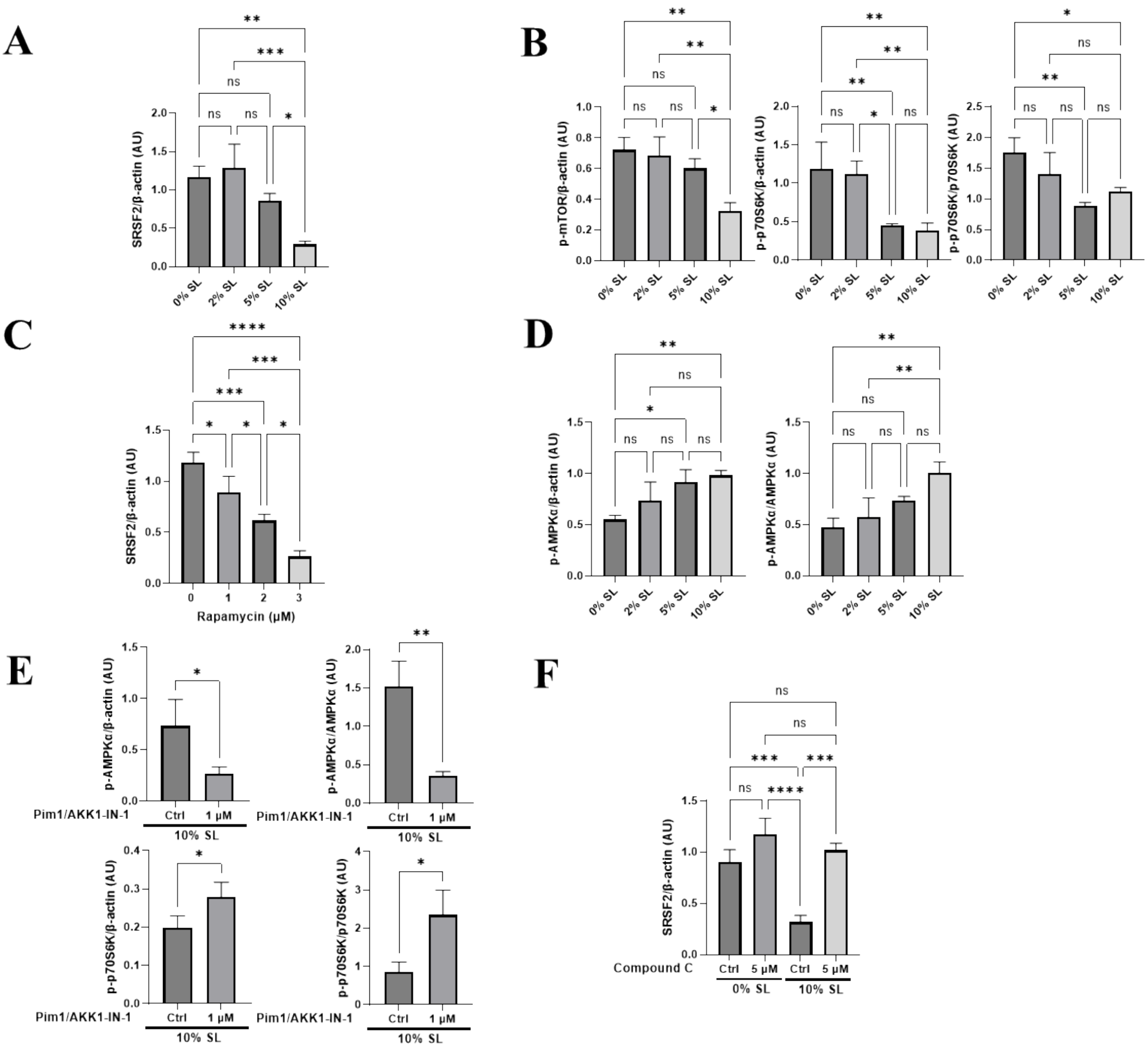

Figure S3. Densitometry data of Western blot analysis corresponding to Figure 3. Quantification of target proteins was normalized to β-actin. In addition, phosphorylation levels of p70S6K and AMPK were normalized to their respective non-phosphorylated forms. (A) Decreased expression of SRSF2 in muscle cells in response to increased intensity of SL. (B) Reduced expression of p-mTOR (Ser2448) and p-p70S6K (Ser371) with increased intensity of SL. (C) Muscle cells treated with 1-3 µM rapamycin for 24 hours. The results exhibit a dose-dependent reduction in SRSF2 expression. (D) Increased expression of pAMPKα (Thr172) in muscle cells following SL. (E) Pim1/AKK1-IN-1 pretreatment abolished AMPK phosphorylation and rescued p70S6K phosphorylation in loaded muscle cells. (F) Effect of 5 µM Compound C (CC) treatment on SRSF2 expression in muscle cells exposed to 10% SL. Muscle cells were incubated with or without CC for 24 hours. The addition of CC rescued the expression of SRSF2 in muscle cells subjected to 10% SL. Statistical significance is indicated as * $p < 0.05$, ** $p < 0.01$, *** $p < 0.001$, **** $p < 0.0001$.

Table S1. Genes that are down-regulated in both 5% and 10% loading groups in muscle cells.

		5% loading			10% loading	
gene_name	log2_fc	p_value	adj_p_value	log2_fc	p_value	adj_p_value
RPSA	-1,28325	2,14E-06	0,002207	-1,51238	1,40E-06	0,000803
SUB1	-1,49683	2,44E-06	0,002207	-1,81225	2,23E-05	0,001081
SUPT5H	-1,39467	2,71E-06	0,002207	-1,29121	4,53E-06	0,000853
SRSF2	-1,16393	3,91E-06	0,002389	-2,11602	3,55E-07	0,000803
RPS21	-1,17807	7,92E-05	0,009215	-1,50877	1,08E-05	0,000969
PPIL4	-1,13463	0,002354	0,028137	-1,49396	0,002392	0,009308

Table S2. Top 10 down-regulated genes after 5% and 10% loading in muscle cells.

gene_name	comparison	log2_fc	p_value	adj_p_value
SUB1	5% loading	-1,496832584	2,4438E-06	0,0022068
SRSF2	5% loading	-1,394668259	2,7111E-06	0,0022068
UFM1	5% loading	-1,36381981	0,00018547	0,0105479
PPP1R8	5% loading	-1,327664147	5,3973E-05	0,00777177
RPSA	5% loading	-1,283253309	2,1419E-06	0,0022068
RPS21	5% loading	-1,178066454	7,9246E-05	0,00921516
GTF2F1	5% loading	-1,172410968	2,4851E-05	0,00606852
SUPT5H	5% loading	-1,163929397	3,9129E-06	0,00238882
NUCKS1	5% loading	-1,140739407	1,3149E-05	0,00455256
PPIL4	5% loading	-1,134630266	0,00235399	0,02813748

gene_name	comparison	log2_fc	p_value	adj_p_value
SRSF2	10% loading	-2,116021121	3,5456E-07	0,00080268
SUB1	10% loading	-1,812245984	2,2275E-05	0,00108108
H3-7	10% loading	-1,79635177	0,01620032	0,03402208
NFYC	10% loading	-1,768613524	4,6575E-06	0,00085332
SRSF11	10% loading	-1,696536298	4,0204E-05	0,00138589
DDX46	10% loading	-1,6766927	2,7496E-05	0,00115382
INTS4	10% loading	-1,54298601	7,8394E-05	0,00185456
PPP4C	10% loading	-1,53449869	0,00021031	0,00258919
RPSA	10% loading	-1,512376762	1,3952E-06	0,00080268
RPS21	10% loading	-1,50876746	1,0778E-05	0,0009692
