# Supplementary material for: Mechanical Loading Modulates AMPK and mTOR Signaling in Muscle Cells
Source: J Proteome Res. 2024 Aug 30;23(10):4286–95. doi: 10.1021/acs.jproteome.4c00242 (PMC11459513; doi:10.1021/acs.jproteome.4c00242)
Supplement: Supplementary file 6 — pr4c00242_si_006.pdf [file pr4c00242_si_006.pdf]

Figure 3A

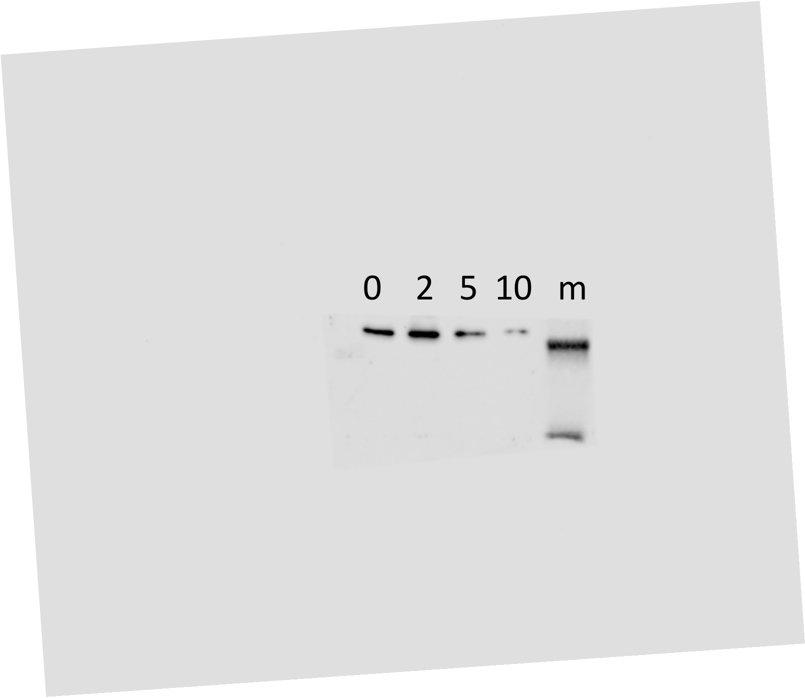

SRSF2

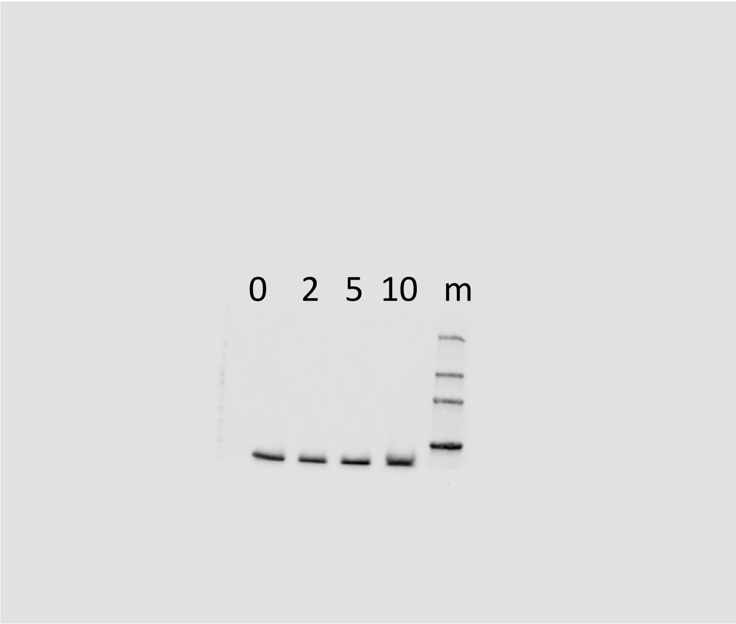

Actin

Figure 3B

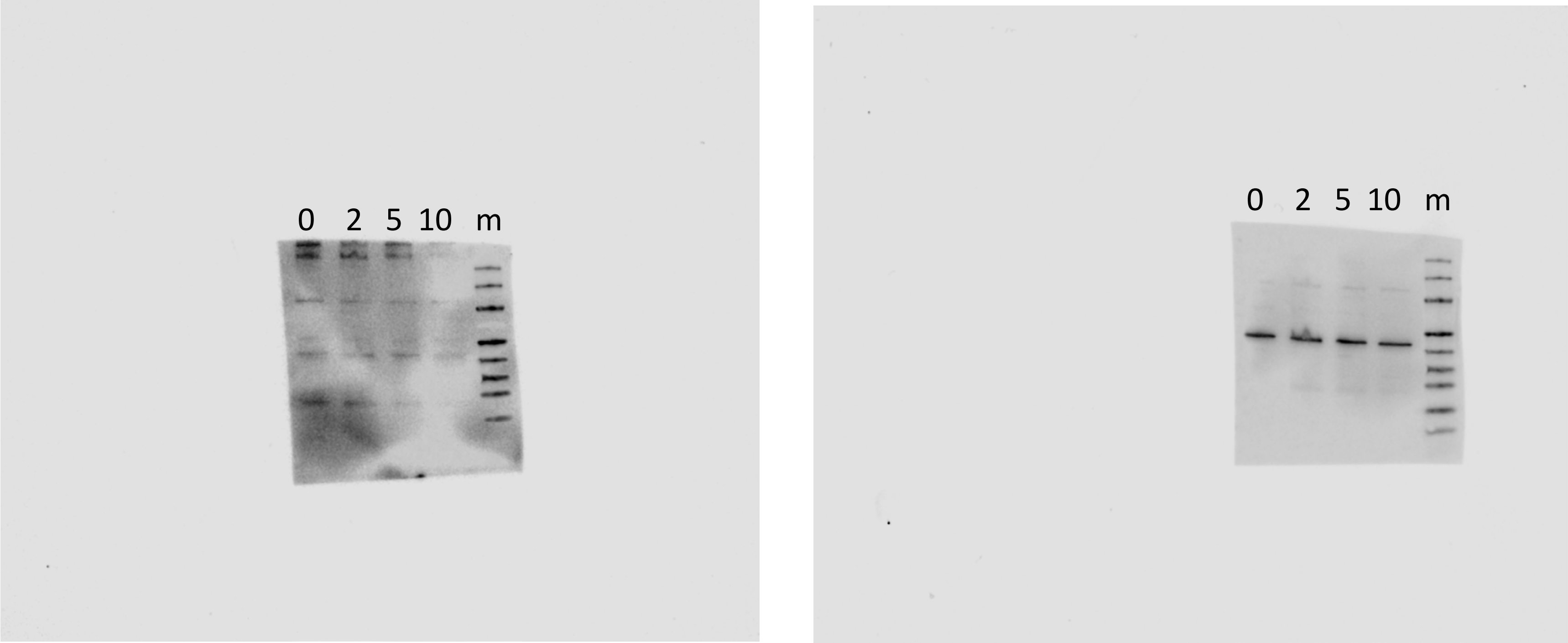

p-mTOR

Actin

Figure 3B

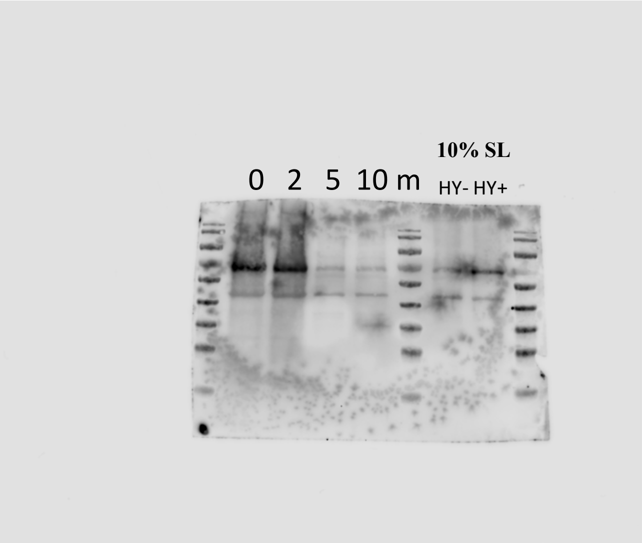

p-p70S6K

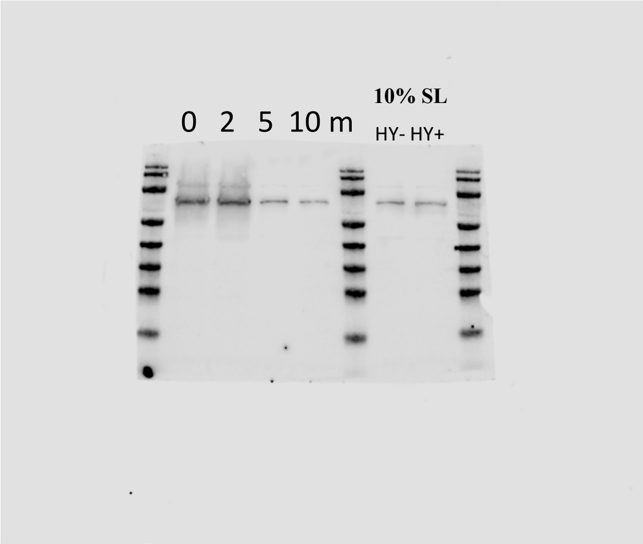

p70S6K

Figure 3C

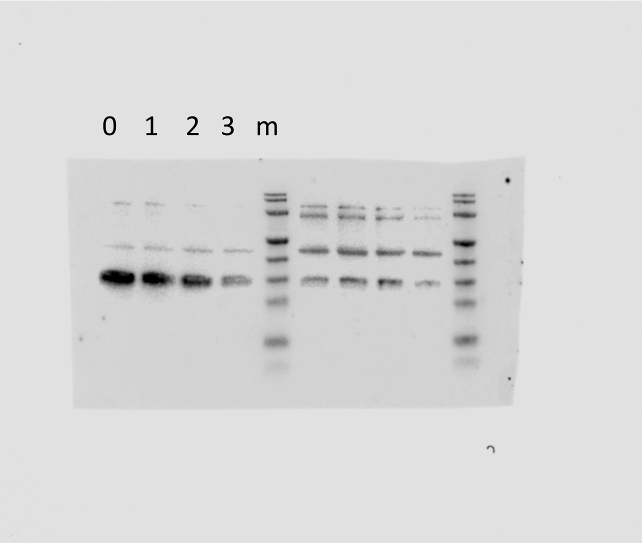

SRSF2

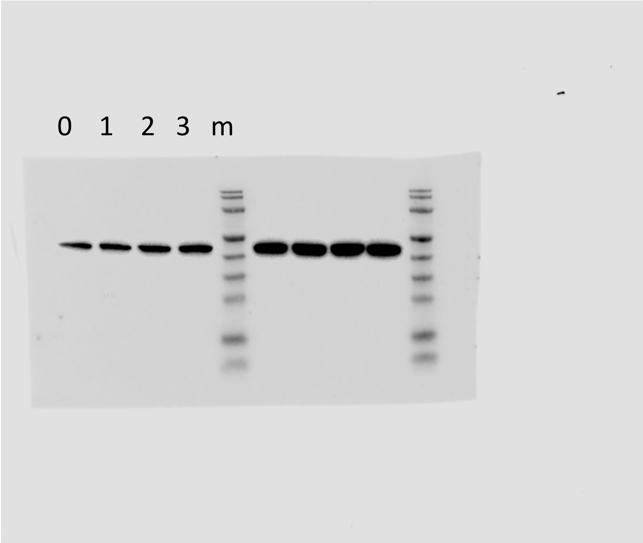

Actin

Figure 3D

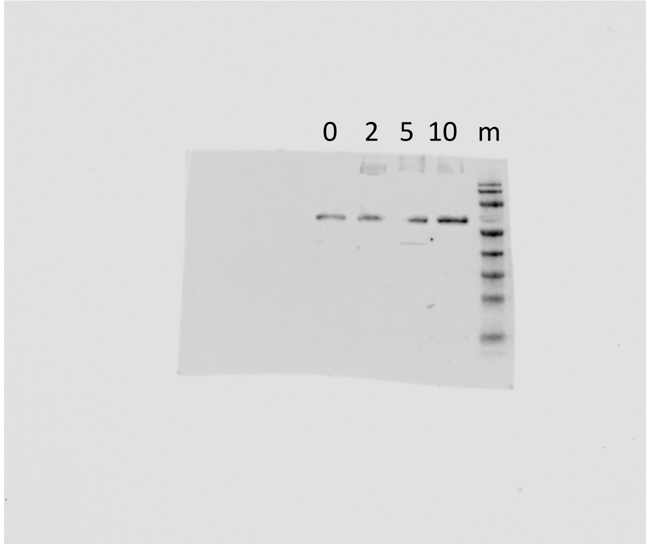

p-AMPK

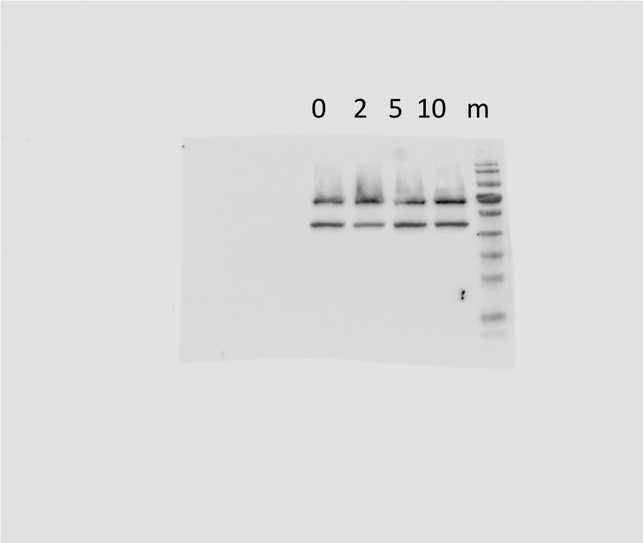

AMPK+actin

Figure 3E

10% SL

HY- HY+

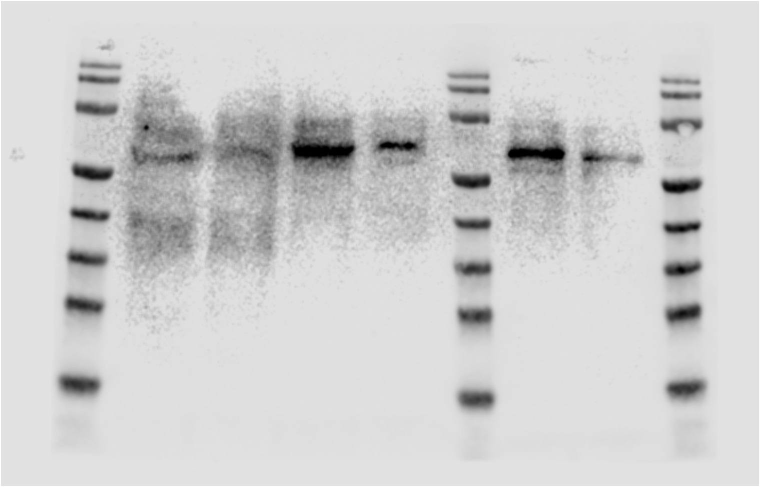

p-AMPK

10% SL

HY- HY+

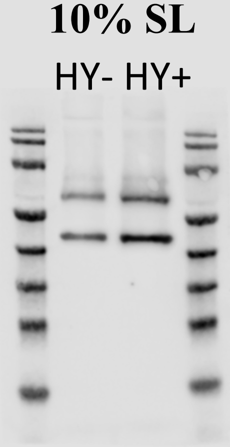

AMPK+actin

Figure 3E

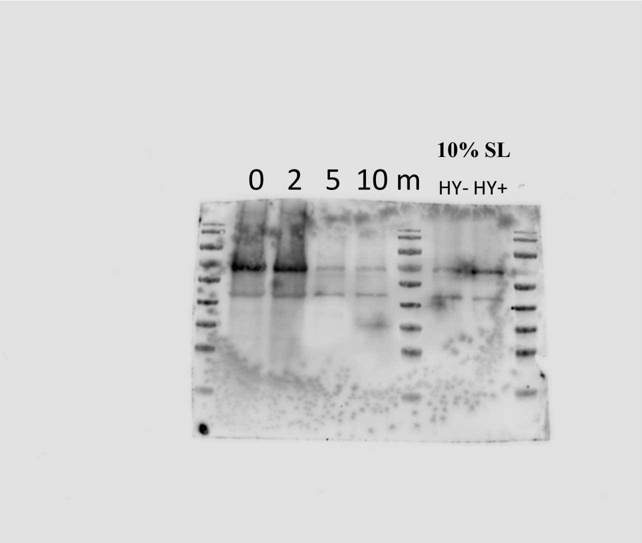

p-p70S6K

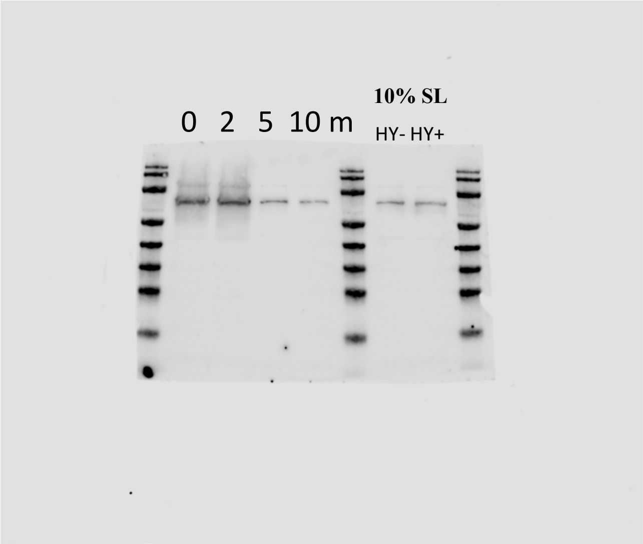

p70S6K

Figure 3F

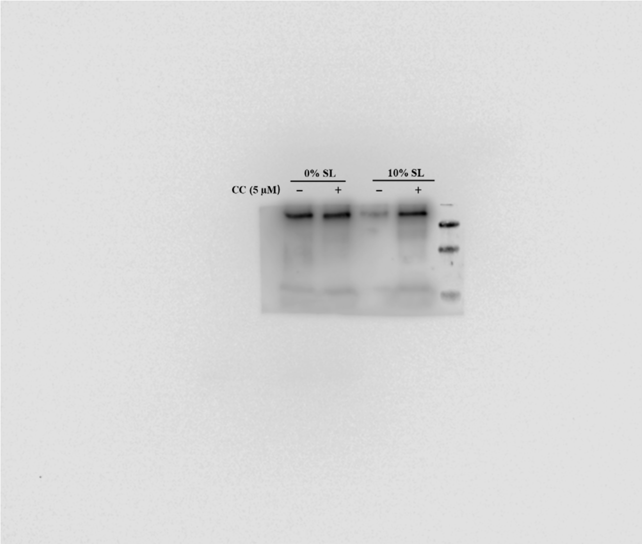

SRSF2

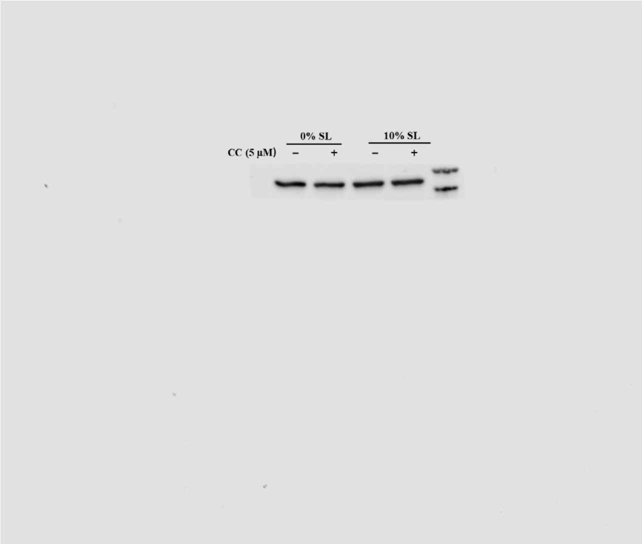

Actin
